# Prophylaxis in Dentistry

**Published:** 1900-12

**Authors:** D. D. Smith


### PROPHYLAXIS IN DENTISTRY.[1]

BY D. D. SMITH, D.D.S., M.D.

It was the privilege of the author to first present the subject-matter of this paper before the Washington City Dental Society, February 24, 1898. The talk then made was afterwards elaborated and presented as a paper before the Northeastern Dental Society at Hartford, Conn., in October of the same year. This paper was published in the International Dental Journal, January, 1899. The theories then timidly advanced having in experimental practice apparently congealed into concrete principles, it seems fitting and appropriate that the matter should be brought in a formal manner to the notice of this Academy, for the significance attaching to the principles herein enunciated should command the most serious attention from the reflective and earnest minds of the profession.

Whilst it is not a written declaration of dentistry, there is nevertheless a distinct impression emanating from dental literature and dental teachings, to the effect that any advancement in dentistry must be either through improved mechanism, new materials, or new methods of operating; in other words, there always has been and still is given to the *mechanics* of dentistry a decided preponderance of energy and effort.

It has been the good fortune of the author, while seeking an interpretation of certain phenomena which are always attendant upon the more common dental lesions, to be led out of the beaten

---

[1] Read before the Philadelphia Academy of Stomatology, June 26, 1900.

paths of thought and effort and into investigations for the betterment of the teeth, in distinctively opposite ways and by distinctively opposing methods. The results of these investigations and experiments, like the methods employed in producing them, are wholly antipodal to the present thought of the profession; hence they are brought to your notice, and thus to the dental profession, with a degree of hesitancy and with some trepidation.

It is my belief and conviction, that when the secrets of the etiology of caries of the teeth shall be clearly unfolded, the inevitable conclusion will be that to *tooth environment* rather than to structural composition, is due by far the larger percentage of dental troubles. In the article on this subject published in the INTERNATIONAL DENTAL JOURNAL, January, 1899, by the author, the *manner* of tooth destruction through decay was fully and, as we believe, for the first time definitely and clearly set forth. Firmly convinced of the impregnable nature of these propositions, my efforts have centred upon means and methods for producing and maintaining the most perfect attainable isolation of the teeth from concomitant agencies of decay.

Special foods and methods of infant-feeding for the production of structural tooth resistance ever have been and must continue practically abortive; and especially so as affecting aggregations or any considerable numbers suffering from defective dentures; hence no investigations were made in this direction. The very pinnacle of absurdity in this field seems to have been attained at a recent dental meeting in Richmond, when, in criticising certain measures for compulsory teachings respecting the teeth and the enforcement of methods of caring for them in the public schools, one gentleman maintained that the wiser and better course would be for dentists to teach physicians (!) the proper methods of attending to the dentition of children; for physicians, having in their charge the "little stomachs" of the children, could prescribe and regulate food for them and thus maintain good digestion and consequently produce good teeth. This incident is introduced here to give emphasis to the impracticability of *any* systematic regulation of children's diet, and because it so plainly illustrates the *reductio ad absurdum* of the proposition that a physician's care or any special feeding will, or can, effect systemic assimilation of special articles of food to the production of structurally good teeth. Given food in proper quantity and in variety such as is ordinarily found in the home of an American family, the system will unerringly appropriate the elements required for tooth-building, through the same elective affinities that obtain in the consolidation of other tissues, and by no other means. The important work devolving on the physician or dentist is to so control environment during the eruptive stage and thereafter (a matter reasonably within the province of either profession), that the formative processes exhibited in the activities of the pulp shall not be hindered or disturbed by adverse chemical agencies acting upon the erupted parts of the tooth.

Dr. Williams, of London, whose writings have been received with considerable favor in this country, in a letter in *Items of Interest,* said, "I have repeatedly pointed out that in my judgment the greatest hope for the future in saving human teeth lies in the direction of the prevention of decay by the use of germicides." The inadequacy of this proposition will be plainly seen when we consider that decay in human teeth is by far the most controllable affection to which they are subject; that bacteria is but one of several causes of decay; and if it were *the* one and only cause of decay and other troubles of the teeth, there has yet been discovered no effective germicide which can be safely used in the mouth. The same writer says further upon this subject: "In my own practice I have relied chiefly upon a strong solution of hydronaphthol in oil of cassia. . . . This I use freely in all cavities, and then before filling I use a varnish of Canada balsam in chloroform in which there is ten per cent. hydronaphthol. My patients use a dentifrice in which hydronaphthol and oil of cassia are the principal germicides. Decay in many instances has been almost entirely arrested." Although Dr. Williams may have relied on these agents in his own practice, until we are given further and more specific information respecting the action of "hydronaphthol in oil of cassia," and some reliable data relating to the efficiency of their germicidal properties, as well as the practicability of their use in dentifrices and mouth-washes, we cannot feel any assurance whatever that in these agents we have any valuable addition to the present long list of so-called germicidal nostrums urgently seeking recognition. Whilst germicidal washes may possibly be made of some value, at present they fall very far short of meeting the requirements for the prevention of tooth decay.

If the generally accepted theories respecting the causes of caries are correct, among which the presence of bacteria and their products stands most prominent, it necessarily follows that *environment* is the important factor governing decay of the teeth; and to systematically enforce a complete and positive change from bad to good in the mouth and about the teeth is a method of prophylaxis both efficient and safe.

The practice conceived, suggested, and instituted by the author, briefly stated, consists in thorough removal at frequent and regular intervals—once every month has thus far proved most satisfactory—of all accumulations, whether solids, inspissated secretions, semisolids, or bacterial formations, from *all* the exposed surfaces of the teeth, leaving the enamel, or whatever of the tooth may be uncovered, thoroughly polished, and thus in the best condition to avoid hurtful deposits and equally to favor all efforts of the patient in the direction of cleanliness.

It is readily demonstrable that to maintain true cleanliness in the mouth, even on the part of the most painstaking, is impracticable, if not impossible, without the direction and assistance of an intelligent and expert operator. There are the calcific deposits, constantly increasing; the more immediately hurtful acidulated bacterial accumulations; inspissated mucus retaining decomposing particles of food and furnishing most favorable conditions for bacterial culture; besides these there are irregularities and certain formations and positions inaccessible to all ordinary methods of cleansing, which implies the perpetual retention of matter inimical to the teeth and gums. These injurious accumulations, with their equally injurious emanations, hitherto overlooked and disregarded by physician or dentist, are not only causes of decay, but are equally causes of recession of gums and absorption of alveolar structure; the latter condition much more to be dreaded than simple decay of the teeth.

Recognition will yet be made of the important fact that to the *presence* rather than to the quantity of foreign matter on and about the teeth the beginnings of pyorrhœa are wholly attributable; and the deleterious influence of a breath perpetually loaded with offensive emanations from this source—especially at seasons of salivary inactivity, as during sleep—will, we believe, ere long be disclosed as an important factor in many pulmonary and digestive disorders, and will be taken account of in medical diagnosis and treatment.

The prophylactic treatment advocated in this paper for the prevention and for the relief of these and other conditions has been found to be not only possible, but its feasibility has been clearly demonstrated. I have described the process more commonly as cleaning the teeth, but there is a wide distinction between the ordinary methods of cleaning the teeth and the system of prophylactic treatment herein contemplated. The difference is: first, in appliances and methods; second, in extent and thoroughness of the operation; third, in the persistence and frequency of the treatment; fourth, in the object sought—the prevention both of decay and pyorrhœa—and in the results attained.

When necessary, scalers should first be used for the removal of solid deposits and such mucous concretions as may have been the means of softening or causing a partial decalcification of cervical enamel. Following this, the teeth should be thoroughly polished on *all* exposed surfaces,—the labial, buccal, palatal, lingual, mesial, distal, and, in cases of developing teeth, the occlusal as well. The hand-polishing with stick and pumice should reach to every exposed portion of the tooth, and be continued until the touch, which can be educated in this matter to distinguish better than the eye, gives evidence of thorough cleansing and polishing. The operation is best done with properly shaped orange-wood sticks charged with powdered pumice-stone. The prepared orange-wood is most conveniently handled and carried to positions desired by means of a Jacke porte-polisher made after a modified pattern given into the hands of J. W. Ivory, of whom they can be purchased.

The grit of the not too finely powdered pumice has been found best adapted for removing viscid, mucoid accumulations and for polishing enamel surfaces; and what is even more important, the friction of the stick and pumice as applied by hand—*for power polishers should never be used*—seems to excite or stimulate the vital forces of the tooth to increased activity in the removal of waste and the deposit of new and better material. The effect seems like massage treatment for muscular tissue.

The benefits resulting from this treatment are marked and readily seen, and extend to all parts of the tooth. Whilst the deciduous and young permanent teeth are most responsive, all classes of teeth and teeth of all ages are peculiarly benefited by the treatment, as is plainly shown in them after a few months of regular and careful massaging. There are striking exhibitions of improvement in color; of change in enamel, from an opaque appearance or condition to that of ivory-like translucency; of apparent increase in density, and a general improvement denoting a condition of decay-resisting structure,—changes which have impressed and even astonished the author as perhaps no other results from operations on the teeth, or in the mouth, have ever done.

Of the about forty cases of all ages which have come regularly under a system of monthly treatment during the past two years, there has been no case of new decay which could not be readily accounted for, neither has there been an instance of beginning pyorrhœa or other pathological condition of the gums. I have under observation now cases of the greatest interest, where the dark yellowish stains in cementum and dentine just above the enamel, and white spots under the enamel, denoting defective nutrition, are beginning to take on a better condition. The yellow is being replaced by better colored material, and the white spots are disappearing, indicative of resumption of normal nutrition. In every case the enamel under this treatment assumes a more pleasing appearance and seems to take on a more vital condition. Erosion is retarded, if not arrested; and the same may be said of that form of wasting which occurs on the labial and buccal surfaces of the teeth, which Dr. Thompson, in an article in the July, 1900, *Dental Digest,* has mistakenly classed under the head of "mechanical abrasion" due to mechanical friction, the result of too vigorous use of the tooth-brush and too gritty tooth-powders. My observation leads to the conclusion that these channels or grooves cut across upper or lower teeth, just at the gum margins, are *never* the result of mechanical abrasion. They appear in the worst form in situations which preclude abrasion from the use of the brush or from any form of friction. They are due to the mucous secretion, intensified in its chemical action by being mechanically held in contact with the teeth by the lips. This condition is found more commonly in mouths where there is limited action or movement of the lips, either in speaking or laughing, thus presenting little opportunity for commingling of mucus and saliva for the neutralization of the acidity of the former.

We need not dwell upon what may be classed as minor benefits resulting from this treatment, although they are as distinct and positive as are the thwarting of tooth decay and the prevention of pyorrhœa. I close with the bare mention of a few of them: Entire and frequent change of environment, resulting in a state of cleanliness of the teeth and mouth unapproached by other means; a breath freed from the emanations of offensive matter persistently on the teeth; perfect physiological gum and substructures; enhanced views of the true importance of the teeth, and the employment of more intelligent and effective methods for ordinary cleansing on the part of the patient; familiarizing children and young people with the dental chair and removing the fear of dental operations; teaching and assisting in the proper care of the teeth at the most critical period of tooth existence; relief from the torturing dread of dental operations, and the fulfilment of the designs of nature, in the comfortable use of the natural teeth through to old age.

